# Efficacy of NEPA, a fixed antiemetic combination of netupitant and palonosetron, vs a 3‐day aprepitant regimen for prevention of chemotherapy‐induced nausea and vomiting (CINV) in Chinese patients receiving highly emetogenic chemotherapy (HEC) in a randomized Phase 3 study

**DOI:** 10.1002/cam4.3123

**Published:** 2020-05-30

**Authors:** Jianhua Chang, Gongyan Chen, Dong Wang, Guihua Wang, Shun Lu, Jifeng Feng, Wei Li, Ping Li, Corinna Lanzarotti, Salvatore Chessari, Li Zhang

**Affiliations:** ^1^ Fudan University Shanghai Cancer Center Shanghai China; ^2^ Affiliated Tumour Hospital of Harbin Medical University Harbin China; ^3^ The Third Affiliated Hospital of Third Military Medical University Chongqing China; ^4^ Changsha Central Hospital Changsha China; ^5^ Shanghai Chest Hospital Shanghai Jiao Tong University Shanghai China; ^6^ Jiangsu Cancer Hospital Nanjing Medical University Affiliated Cancer Hospital Nanjing China; ^7^ The First Affiliated Hospital of Jilin University Changchun China; ^8^ West China School of Medicine West China Hospital Sichuan University Chengdu China; ^9^ Helsinn Healthcare Lugano Switzerland; ^10^ Sun Yat‐sen University Cancer Center Guangzhou China

**Keywords:** antiemetic, aprepitant, CINV, NEPA, palonosetron

## Abstract

NEPA is the only fixed combination antiemetic, comprised of an NK_1_RA (netupitant) and a 5‐HT_3_RA (palonosetron). In the first head‐to‐head trial to compare NK_1_RA‐containing regimens, a single oral dose of NEPA was non‐inferior to a 3‐day aprepitant/granisetron (APR/GRAN) regimen for the primary endpoint of overall (0‐120 hours) complete response (no emesis/no rescue). This pre‐specified analysis evaluates the efficacy of NEPA versus APR/GRAN in the subset of Chinese patients in the study. In addition, efficacy in patients at greatest emetic risk receiving high‐dose cisplatin (≥70 mg/m^2^) was explored. Chemotherapy‐naïve patients with solid tumors in this randomized, double‐blind study received either a single dose of NEPA prior to cisplatin‐based chemotherapy or a 3‐day regimen of APR/GRAN, both with dexamethasone on Days 1‐4. Efficacy was evaluated through complete response, no emesis, and no significant nausea rates during the acute (0‐24 hours), delayed (25‐120 hours) and overall phases as well as individual days post‐chemotherapy, as the daily course of CINV protection is often unstudied. The Chinese subset included 667 patients; of these, 363 (54%) received high‐dose cisplatin. Baseline characteristics were comparable. While response rates were similar for NEPA and APR/GRAN during the acute, delayed and overall phases, significantly fewer NEPA patients experienced breakthrough CINV on individual Days 3‐5 in both the Chinese patients and also in those receiving high‐dose cisplatin. As a fixed oral NK_1_RA/5HT_3_RA combination given once/cycle, NEPA is a convenient highly effective prophylactic antiemetic that may offer better protection from CINV than a 3‐day APR/GRAN regimen on Days 3‐5 following highly emetogenic chemotherapy.

## Graphical abstract

NEPA is a novel fixed combination antiemetic comprised of the NK_1_RA, netupitant, and the 5‐HT_3_RA, palonosetron. The simultaneous targeting of two critical antiemetic pathways, in unison with single‐dose administration, offers a simplified and convenient antiemetic with 5‐day CINV prevention. The authors report significantly better protection from CINV during Days 3‐5 post‐chemotherapy for NEPA vs a 3‐day regimen of aprepitant/granisetron in Chinese patients, and in those receiving high‐dose cisplatin, in the first head‐to‐head study comparing NK_1_RA‐containing regimens.

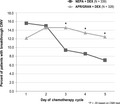

## 1 INTRODUCTION

Continual advances in the antiemetic supportive care field of oncology have led to dramatic improvements in the prevention of chemotherapy‐induced nausea and vomiting (CINV), as recognized by the American Society of Clinical Oncology (ASCO) who acknowledged the development of effective antiemetic therapies among the top five advances in oncology over the last 50 years following a world‐wide survey.^1^, ^2^ With administration of guideline‐recommended antiemetic prophylaxis, CINV can now be prevented in most patients.^3^, ^4^ As a result, the quality‐of‐life of cancer patients has substantially improved, allowing patients to be treated without chemotherapy disruption or dose reductions caused by these side effects.^1^

Evidence‐based antiemetic guidelines^5^, ^6^, ^7^, ^8^ now consistently recommend multi‐agent prophylactic combinations that target different receptors involved in the CINV process. Guidelines also provide details on the dose and administration frequency of these agents. For patients receiving highly emetogenic chemotherapy (HEC), co‐administration of a triplet antiemetic regimen of a neurokinin‐1 (NK_1_) receptor antagonist (RA), a 5‐hydroxytryptamine‐3 (5‐HT_3_) RA, and dexamethasone (DEX) is recommended,^5^, ^6^, ^7^, ^8^ ± a fourth agent, olanzapine.^5^, ^6^

Unfortunately, antiemetic guidelines are often not adhered to in clinical practice, despite evidence confirming that CINV control is optimized when recommendations are followed.^3^, ^4^ It is possible that the complexity of these multimodal antiemetic regimens contributes to this nonadherence, as each of the components of the regimen may have differing formulations, doses, and schedules that are to be given prophylactically from the day of chemotherapy to 4 days post‐chemotherapy. Simpler, less frequent dosing regimens may improve compliance.^9^

Oral NEPA is the only fixed antiemetic combination agent. It is comprised of the highly selective NK_1_RA, netupitant (300 mg), and the clinically and pharmacologically^10^ distinct 5‐HT_3_RA, palonosetron (0.5 mg). The simultaneous targeting of two critical antiemetic pathways, in unison with single‐dose administration, offers a simplified and convenient antiemetic with 5‐day CINV prevention.

Studies supporting the registration of oral NEPA in the United States (US) and Europe (EU)^11^, ^12^ showed superiority of oral NEPA plus DEX over oral palonosetron plus DEX in preventing CINV during the acute (0‐24 hours), delayed (25‐120 hours), and overall (0‐120 hours) phases following both cisplatin‐^13^ and anthracycline‐cyclophosphamide (AC)‐based chemotherapy.^14^, ^15^ In addition, NEPA was shown to be efficacious over multiple cycles in patients receiving either HEC or moderately emetogenic chemotherapy (MEC).^16^ The overall results of a Phase 3 study designed in collaboration with the China Food & Drug Administration [CFDA, which became known as the National Medicinal Products Administration (NMPA) in 2018] formed the basis for the recent registration of oral NEPA in China and have been previously published.^17^ This study was conducted in Asia and evaluated the efficacy of a single dose of oral NEPA plus DEX compared with a standard 3‐day regimen of aprepitant plus granisetron (APR/GRAN) plus DEX, in patients receiving cisplatin‐based HEC. Non‐inferiority of single‐dose NEPA to 3‐day APR/GRAN was shown for the primary efficacy endpoint of overall complete response (no emesis/no rescue medication). Per registration requirements in China, this regional study conducted in Asia was designed to support the recent approval of oral NEPA in China. As part of this regulatory requirement, a prospective analysis was required evaluating the efficacy of NEPA compared with the APR/GRAN regimen in the subset of Chinese patients enrolled; as was the case for the overall study population, the hypothesis was non‐inferiority of NEPA and APR/GRAN. This paper presents these findings in this Chinese subset.

Cisplatin is well established as the most emetogenic HEC agent, with antiemetic trials demonstrating a dose‐dependent effect on emesis control.^18^, ^19^, ^20^ Therefore, a post hoc evaluation of the efficacy of oral NEPA in Chinese patients receiving the highest doses of cisplatin (≥70 mg/m^2^) (ie, those presumed to be at the greatest emetic risk) was of interest and is also presented.

## 2 PATIENTS AND METHODS

### 2.1 Study design

This was a Phase 3, multicenter, randomized, double‐blind/double‐dummy, single cycle, international study registered with the China Registration and Information Disclosure Platform for Drug Clinical Studies (Registration Number CTR20130417). Patients were randomized between February 2014 and August 2015 at 46 enrolling sites in four countries, with the majority of sites in China (ie, 30 in China, five Taiwan, three Thailand, and eight Korea).

The trial protocol (Protocol Synopsis, Supplementary Information) was approved by the institutional review board/independent ethics committees and all patients provided written informed consent prior to treatment initiation. The study was conducted in compliance with the Code of Ethics for the NMPA, Good Clinical Practice, the principles of the Declaration of Helsinki, and the International Conference on Harmonization guidelines.

### 2.2 Patients

Inclusion/exclusion criteria were similar to those in the original oral NEPA pivotal trials^13^, ^14^, ^16^ and were presented in detail in the overall study publication.^17^ Key eligibility criteria were that patients were ≥18 years, naïve to chemotherapy, and scheduled to receive their first course of cisplatin‐based (≥50 mg/m^2^) chemotherapy (as monotherapy or in combination with other chemotherapy) for the treatment of a confirmed solid tumor malignancy. Patients were required to have an Eastern Cooperative Oncology Group (ECOG) Performance Status of 0‐2.

Patients were not eligible if they were scheduled to receive: (a) MEC or HEC from Days 2‐5 following cisplatin, (b) moderately or highly emetogenic radiotherapy within 1 week prior to Day 1 or between Days 1 and 5, or (c) a bone marrow or stem‐cell transplant.

### 2.3 Treatment

Treatment assignment was managed through static central blocked randomization, stratified by gender, using an interactive web response system (IWRS). The randomization scheme was reproducible and was prepared by the contract research organization via a computerized system prior to the start of the study. The IWRS provider retained master copies of the randomization codes in a secure fashion in order to maintain the blind. Considering gender as a stratification factor, patients who met the inclusion and exclusion criteria were assigned to one of the two treatment arms (either NEPA or APR/GRAN treatment) in a balanced fashion (1:1), using the randomization system and according to specific procedures.

On Day 1, oral NEPA and oral APR 125 mg were administered 60 minutes and IV GRAN 3 mg and oral DEX 12 mg were administered 30 minutes prior to chemotherapy, respectively (Supplementary Table S1). APR 80 mg was also administered 24 and 48 hours (on Days 2 and 3) after its administration on Day 1. On Days 2‐4, DEX 8 mg was administered in both groups. The DEX dose was consistent with that specified in the product labeling for both NEPA and APR, and the 3‐mg GRAN dose is the registered dose in China. Due to the blinded, double‐dummy study design, placebos fully matching in appearance to 1) oral NEPA, 2) oral aprepitant, and 3) IV granisetron were required.

### 2.4 Assessments and endpoints

During the 0‐120 hours 5‐day period post‐chemotherapy, each patient completed a diary, capturing emetic episodes, severity of nausea, and concomitant medications, including rescue. Severity of nausea was evaluated using a 100‐mm horizontal visual analog scale (VAS) ranging from “no nausea” (0 mm) to “nausea as bad as it could be” (100 mm). The Functional Living Index‐Emesis (FLIE) questionnaire [nine nausea‐specific (nausea domain) and nine vomiting‐specific (vomiting domain) items] was used to assess the impact of CINV on patient's daily life. Responses were marked on a 100‐mm VAS with anchors of 1 and 7. Patients completed this questionnaire on Days 2 and 6 assessing the impact of CINV on their lives on Day 1 and during Days 2‐5.^21^

Efficacy endpoints included assessment of complete response, no emesis, and no significant nausea (defined as VAS score <25 mm) during the acute (0‐24 hours, Day 1), delayed (25‐120 hours, Days 2‐5), and overall (0‐120 hours, Days 1‐5) phases as well as daily during the 0‐120 hours period post‐chemotherapy. The primary endpoint in this Chinese population was complete response in the overall phase, corresponding to the primary endpoint of the study for the overall study population.^17^ Additional secondary endpoints of daily rates of breakthrough CINV (ie, proportions of patients experiencing emesis and/or using rescue medication, reflective of those patients who did not experience complete response) were calculated for the overall Chinese population and also for the subset of patients receiving high‐dose cisplatin. For the FLIE assessment, the proportion of patients with scores reflecting “no impact on daily life” (NIDL) (ie, individual question scores >6 on the 7‐point FLIE scale, domain score >54, and overall FLIE score >108) was evaluated as a secondary “quality‐of‐life” endpoint.

Safety was assessed by collection of adverse events, vital signs, physical examination, clinical laboratory tests, and electrocardiograms (predose, and 5, 24, and 120 hours post‐dose).

### 2.5 Statistical analysis

Assessment of efficacy in the Chinese population was a prespecified subset analysis and included an adequate number of patients based on requirements from the Chinese NMPA. The frequency counts and percentages of patients achieving all endpoints described above were estimated and summarized by treatment group. The risk difference for NEPA – APR/GRAN and associated 95% confidence intervals were analyzed using the Cochran‐Mantel‐Haenszel (CMH) test using gender as a stratum in the model. Adjustment for multiple comparisons on secondary endpoints was not performed.

Assessment of efficacy in the Chinese population receiving the highest doses of cisplatin (≥70 mg/m^2^) (ie, those patients at the greatest risk for CINV) was a post hoc analysis. The same descriptive approach to evaluation of the efficacy endpoints in this subset was taken.

The number and proportion of patients who experienced treatment‐emergent adverse events (AEs) and treatment‐related adverse events (TRAEs) were listed and summarized by treatment group. The full analysis set (FAS) population (efficacy analyses) was defined as all patients who were randomized and received protocol‐required cisplatin and study treatment. The safety analysis population consisted of all patients who received study treatment.

## 3 RESULTS

### 3.1 Analyzed patient population

Of the 834 patients randomized into the study, 672 (80.6%) were from China. Three patients and one patient randomized to NEPA and APR/GRAN, respectively, did not receive study treatment and were therefore excluded from the safety population and an additional NEPA‐treated patient did not receive the protocol‐required HEC and was therefore also excluded from the FAS population. Consequently, 668 (340 NEPA/328 APR/GRAN) and 667 (339 NEPA/328 APR/GRAN) represented the safety and FAS efficacy populations, respectively.

Baseline characteristics were similar between treatment groups (Table 1). The Chinese population was predominantly males (69.3%); lung cancer was the most common (72.4%) cancer type.

**TABLE 1:** Patient baseline and disease characteristics (FAS population)

Characteristic	NEPA + DEX	APR/GRAN + DEX
	(N = 339)	(N = 328)
Gender		
Male	234 (69.0%)	228 (69.5%)
Female	105 (31.0%)	100 (30.5%)
Age (y), Mean (SD)	54.4	54.9
ECOG Performance Status		
0	132 (38.9%)	123 (37.5%)
1	201 (59.3%)	197 (60.1%)
2	6 (1.8%)	8 (2.4%)
Most Common (≥5%)		
Cancer Types		
Lung	250 (73.8%)	233 (71.0%)
Cisplatin		
Dose <70 mg/m^2^	149 (44.0%)	155 (47.3%)
Dose ≥70 mg/m^2^	190 (56.0%)	173 (52.7%)
Most Common (≥5%) Concomitant Chemotherapy		
Gemcitabine/Gemcitabine HCl	108 (31.9%)	78 (23.8%)
Pemetrexed/Pemetrexed disodium	63 (18.6%)	71 (21.6%)
Docetaxel	62 (18.3%)	64 (19.5%)
Etoposide	55 (16.2%)	48 (14.6%)

Abbreviations: APR, aprepitant; ECOG, Eastern Cooperative Oncology Group; GRAN, granisetron; SD, standard deviation.

### 3.2 Efficacy

#### 3.2.1 All Chinese patients

**FIGURE 1:**
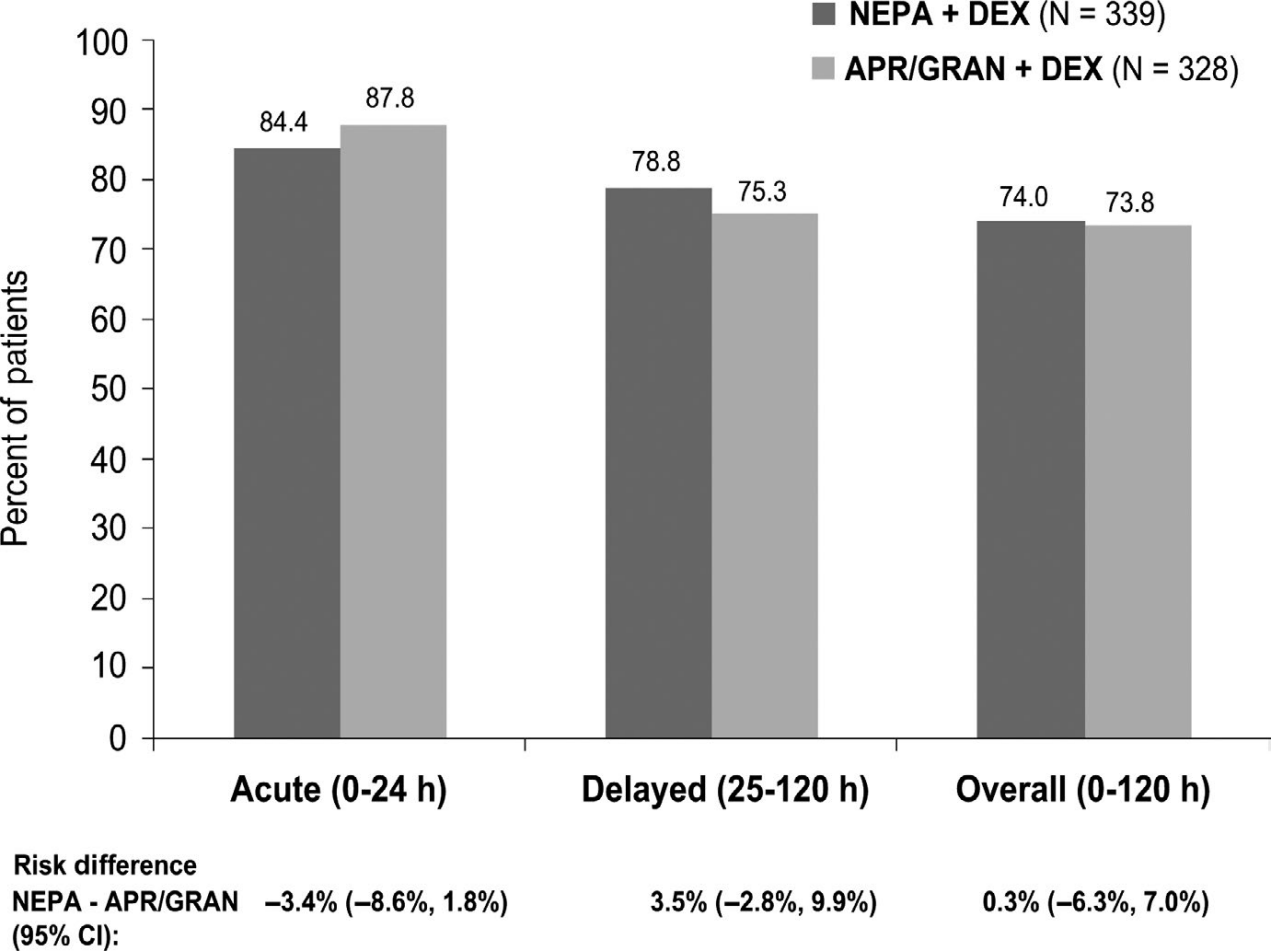
Complete response rates

Complete response rates were similar for oral NEPA and APR/GRAN during the acute, delayed, and overall phases (Figure 1). Similar results were seen for the proportion of patients with no emesis and for those with no significant nausea (Table 2).

**TABLE 2:** No emesis and no significant nausea rates during individual Days 1‐5

Endpoint	NEPA + DEX	APR/GRAN + DEX	Risk Difference	*P*‐value
% Patients	(N = 339)	(N = 328)	NEPA‐APR/GRAN (95% CI)	
No Emesis				
Acute (Day 1)	85.3%	88.1%	−2.8% (−7.9%. 2.3%)	.282
Day 2	85.5%	86.6%	−1.0% (−6.2%, 4.2%)	.711
Day 3	91.2%	85.7%	5.5% (0.7%, 10.3%)	.025
Day 4	92.0%	88.1%	3.9% (−0.6%, 8.5%)	.089
Day 5	93.5%	88.1%	5.4% (1.0%, 9.8%)	.015
Delayed (Days 2‐5)	80.2%	77.4%	2.9% (−3.3%, 9.0%)	.362
Overall (Days 1‐5)	75.2%	75.6%	‐0.3% (−6.8%, 6.2%)	.922
No Significant Nausea				
Acute (Day 1)	89.1%	88.1%	1.0% (−3.8%, 5.8%)	.684
Day 2	86.1%	82.6%	3.6% (−1.9%, 9.0%)	.204
Day 3	87.6%	82.0%	5.6% (0.2%, 11.1%)	.043
Day 4	87.0%	82.6%	4.4% (−1.0%, 9.9%)	.113
Day 5	86.7%	81.4%	5.3% (−0.2%, 10.9%)	.059
Delayed (Days 2‐5)	77.9%	73.8%	4.1% (−2.4%, 10.6%)	.212
Overall (Days 1‐5)	74.9%	71.0%	3.9% (−2.8%, 10.7%)	.252

Abbreviations: APR, aprepitant; DEX, dexamethasone; GRAN, granisetron.

**FIGURE 2:**
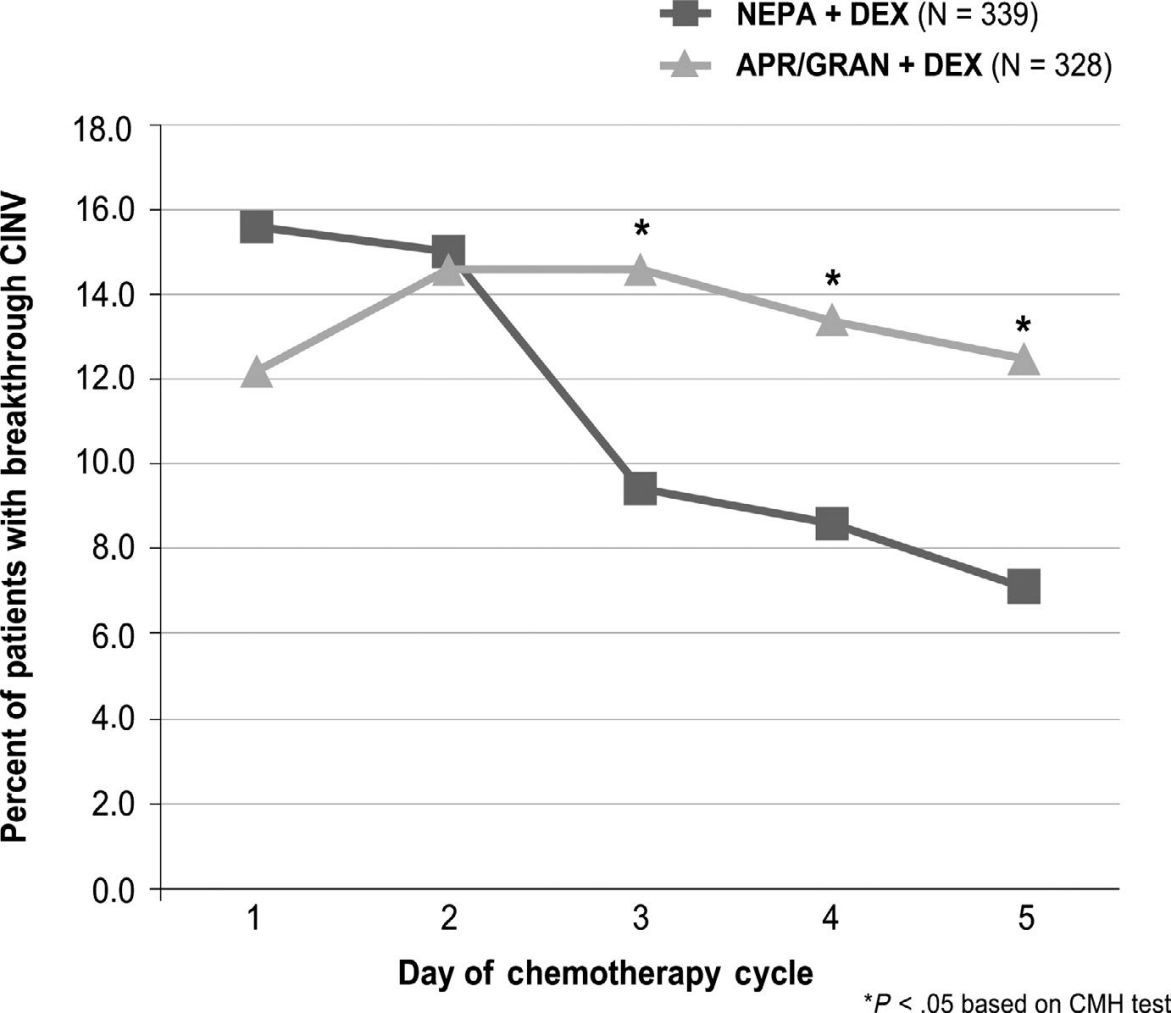
Percent of patients with breakthrough CINV on Days 1‐5

While daily rates of breakthrough CINV remained stable for APR/GRAN over time (12.2%‐14.6%), rates declined from 15.6% to 7.1% over the 5 days for NEPA, with significantly fewer NEPA patients experiencing breakthrough CINV on Days 3‐5 (Figure 2).

Likewise, daily no emesis and no significant nausea rates were similar for NEPA and APR/GRAN on Days 1‐2 and favored NEPA on Days 3‐5, with statistical significance seen for no emesis on Days 3 and 5 and for no significant nausea on Day 3 (Table 2).

**FIGURE 3:**
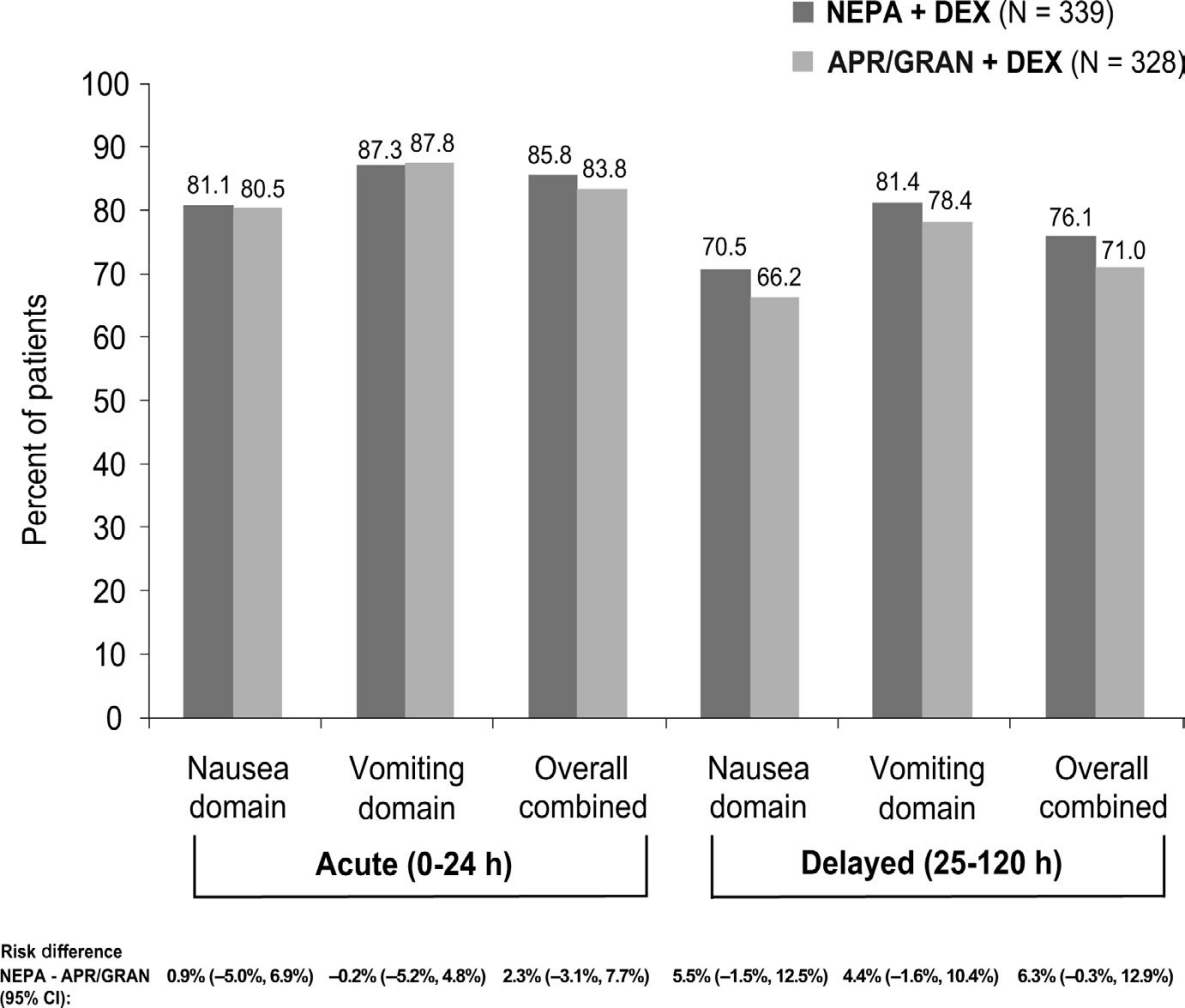
Proportion of Chinese patients with no impact on daily living (NIDL) based on the functional living index‐emesis (FLIE)

The proportion of patients reporting NIDL due to nausea (nausea domain), vomiting (vomiting domain), or both (overall domain) was similar for NEPA and APR/GRAN during the acute phase and numerically favored NEPA during the delayed phase (Figure 3).

#### 3.2.2 Chinese patients receiving ≥70 mg/m^2^ cisplatin

**FIGURE 4:**
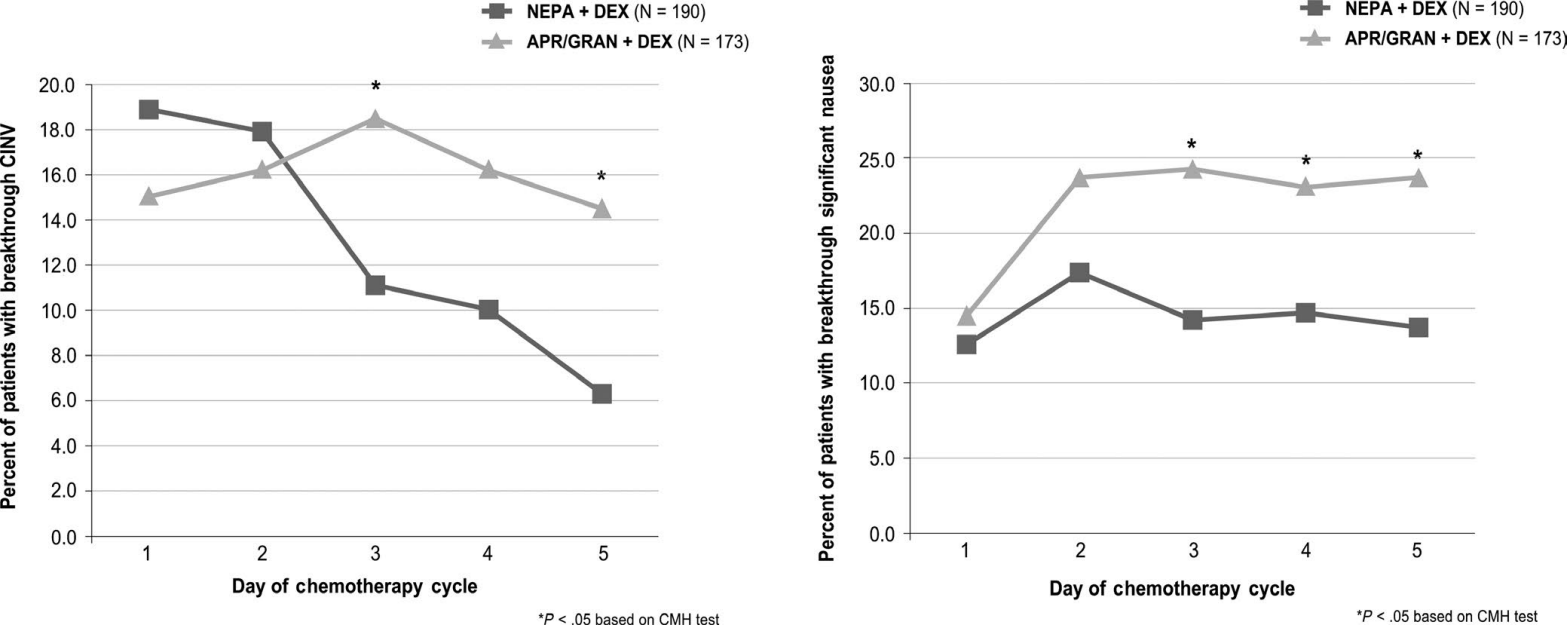
A, Percent of patients with breakthrough CINV on Days 1‐5: high dose (≥70 mg/m^2^) cisplatin subset. B, Percent of patients with breakthrough significant nausea on Days 1‐5: high dose (≥70 mg/m^2^) cisplatin subset

In the subset of patients receiving the highest doses of cisplatin, fewer NEPA patients experienced breakthrough CINV than those in the APR/GRAN group on Day 3 (11.1% vs 18.5%, *P* = .04), Day 4 (10.0% vs 16.2%, *P* = .08), and Day 5 (6.3% vs 14.5%, *P* = .01) (Figure 4A). Similar results were seen for breakthrough significant nausea on Day 3 (14.2% vs 24.3%, *P* = .01), Day 4 (14.7% vs 23.1%, *P* = .04), and Day 5 (13.7% vs 23.7%, *P* = .01) for NEPA vs APR/GRAN, respectively (Figure 4B).

#### 3.2.3 Safety

The incidence of AEs in the Chinese population was very similar between the two treatment groups (NEPA 53.5%, APR/GRAN 53.0%). The most common TRAE was constipation (NEPA 8.8%, APR/GRAN 7.0%); all others had an incidence rate of ≤2%. Among the patients reporting AEs, the majority (91.3%) reported events of mild/moderate intensity, with more severe AEs occurring in the APR/GRAN group (10.4% vs NEPA 7.1%). There were two NEPA patients and one APR/GRAN patient with serious TRAEs (NEPA: 1) atrial fibrillation and 2) chest pain/hypotension/decreased heart rate/nonresponsiveness (concomitant medication included amifostine); APR/GRAN: increased alanine aminotransferase). While the patient experiencing the chest pain/hypotension event recovered within an hour of NEPA treatment, this was the only AE leading to discontinuation from study; two patients treated with APR/GRAN discontinued from the study due to unrelated AEs. There were no deaths in the NEPA group, while two patients treated with APR/GRAN died due to unrelated AEs. Changes from baseline in 12‐lead ECGs were rare and similar.

## 4 DISCUSSION

As a requirement for registration in China, the CFDA necessitated that NEPA demonstrate comparable efficacy to a guideline‐recommended NK_1_RA‐containing triplet regimen.^22^ Thus, this Phase 3 study in Asian patients receiving cisplatin‐based HEC was designed to and subsequently demonstrated non‐inferiority of a single dose of oral NEPA (plus DEX) to a standard 3‐day aprepitant/granisetron/DEX regimen for the primary endpoint of overall complete response.^17^ For secondary efficacy endpoints of no emesis and no significant nausea, delayed and overall rates were numerically but not significantly higher for NEPA. Significantly more NEPA patients did not use any rescue medication during the delayed and overall phases. Antiemetic trials typically evaluate CINV control during acute, delayed, and overall phases post‐chemotherapy; however, the daily course of CINV control is often unstudied beyond Day 1 (the acute phase). In this study, daily rates during the 5‐day overall phase were a prespecified secondary endpoint and can be viewed as either control rates or the inverse rates of “breakthrough” CINV. In the overall study population, daily rates of CINV “events” (ie, emesis and/or rescue use) did not change substantially throughout Days 1‐5 for APR/GRAN; however, rates for NEPA declined numerically over time and were significantly lower than APR/GRAN on Day 5, suggesting a benefit of NEPA during the delayed period post‐chemotherapy.

In follow‐up, the aim of the current paper was to describe the results of a prespecified analysis examining the efficacy of NEPA vs APR/GRAN in the large subset of Chinese patients enrolled in this study. As Chinese patients represented approximately 80% of the overall study population, it was not surprising that the efficacy results in this subset were consistent with the findings of the overall study. Of most interest were the daily response rates (or the inverse reflecting breakthrough CINV rates) where the trends seen for the overall study were also apparent in this subset, with an incremental absolute benefit of NEPA over APR/GRAN of approximately 5% of patients for all endpoints during Days 3‐5. These differences reached statistical significance for breakthrough CINV (emesis/rescue use) (*P* < .05 on Days 3‐5), no emesis (*P* < .05 Days 3 and 5), and no significant nausea (*P* < .05 on Day 5). Also consistent with the overall study results, the higher response rates for NEPA during the delayed phase were reflected in a potential quality‐of‐life benefit, with a correspondingly numerically greater proportion of NEPA patients with no impact on their functioning due to nausea and vomiting during this period when CINV control is typically most challenging.

Cisplatin is well established as the most emetogenic HEC agent^18^ and is the most common setting for trials evaluating antiemetic efficacy. There is evidence of a cisplatin dose‐dependent effect on emesis control,^18^, ^19^, ^20^ thereby putting patients receiving higher doses of cisplatin at an increased emetic risk. Considering this, evaluation of the efficacy of NEPA in Chinese patients receiving the highest doses of cisplatin (≥70 mg/m^2^) was of interest and pursued as a post hoc analysis of this subset. Interestingly, in this higher risk subset, the benefits of NEPA were augmented, with an incremental benefit of NEPA over APR/GRAN of 6%‐8% for proportions of patients with breakthrough CINV and 8%‐10% for those with breakthrough significant nausea on Days 3‐5. Despite the smaller sample size in this group, these differences were statistically significant on each day but Day 4 for breakthrough CINV. Exploration of the potential benefit of NEPA over APR/GRAN in this at‐risk population should be examined in a prospective trial powered for this analysis.

The daily control rates of >85% in all NEPA‐treated Chinese patients as well as those receiving high‐dose cisplatin, combined with the evidence suggesting improved control of nausea for NEPA over APR/GRAN, are encouraging, as nausea prevention remains the greatest unmet need in the goal to avoid CINV in all patients undergoing emetogenic chemotherapy. Thus far, the role of other NK_1_RAs in improving nausea control has been unclear with inconsistent results in NK_1_ trials assessing nausea prevention.^23^, ^24^, ^25^, ^26^

Consistent with the overall study results, NEPA was well tolerated with a comparable adverse event profile to APR/GRAN in these Chinese patients. The majority of AEs were mild/moderate in intensity, unrelated to study treatment, and typical for a cancer population undergoing chemotherapy. There were no cardiac safety concerns for either treatment.

While use of the guideline‐recommended multidrug antiemetic combinations has dramatically improved the prevention of CINV, it comes with the complexity of administering antiemetics with different doses, schedules, and formulations. This complexity may contribute to the poor adherence seen in studies examining adherence with antiemetic guideline recommendations.^3^, ^4^ In the current study, the standard aprepitant regimen required co‐administration of oral APR given 60 minutes prior to chemotherapy on Day 1 and again at 24 and 48 hours later along with intravenous GRAN administered 30 minutes prior to chemotherapy on Day 1. Dexamethasone was given on Days 1‐4. Simplifying these regimens may be appealing to clinicians and patients and may enhance compliance with antiemetic guideline recommendations. As a combination of an NK_1_RA, netupitant, and 5‐HT_3_RA, palonosetron, NEPA conveniently packages two classes of antiemetics in a single oral dose administered 60 minutes prior to chemotherapy on Day 1.

In conclusion, this study exploring the efficacy of oral NEPA, a convenient and simplified combination antiemetic, in Chinese patients revealed that single‐dose NEPA more effectively prevents CINV than a 3‐day aprepitant/granisetron regimen during the latter days (3‐5) post‐chemotherapy.

## CONFLICT OF INTEREST

C. Lanzarotti and S. Chessari disclose that they are employees of Helsinn Healthcare. The remaining authors have nothing to disclose.

## AUTHOR CONTRIBUTIONS

Li ZHANG was responsible for the conception and study design. Jianhua CHANG, Gongyan CHEN, Dong WANG, Guihua WANG, Shun LU, Jifeng FENG, Wei LI, Ping LI, and Li ZHANG were responsible for the collection and assembly of data and Corinna Lanzarotti and Salvatore Chessari were responsible for the data analysis and interpretation. All authors were involved in critically reviewing and revising the manuscript, have read and approved the final manuscript, and agree to be accountable for all aspects of the work.

## Supporting information

Supplementary Material

Supplementary Material

## Data Availability

Data will be made available upon reasonable request.
