# Supplementary material for: Efficacy of NEPA, a fixed antiemetic combination of netupitant and palonosetron, vs a 3‐day aprepitant regimen for prevention of chemotherapy‐induced nausea and vomiting (CINV) in Chinese patients receiving highly emetogenic chemotherapy (HEC) in a randomized Phase 3 study
Source: Cancer Med. 2020 May 30;9(14):5134–42. doi: 10.1002/cam4.3123 (PMC7367622; doi:10.1002/cam4.3123)
Supplement: Supplementary file 1 — Supplementary Material [file CAM4-9-5134-s001.docx]

**Supplementary Table: Treatment Groups**

|  | **NEPA Regimen** | | **APR/GRAN Regimen** | | |
| --- | --- | --- | --- | --- | --- |
|  | **Oral NEPA** | **Oral DEX** | **Oral APR** | **IV GRAN** | **Oral DEX** |
| **Day 1** | NEPA capsule (NETU 300 mg/ PALO 0.50 mg) + placebo aprepitant capsule +  placebo GRAN ampoules | 12 mg  (3 4-mg tablets) | 125 mg APR capsule + placebo NEPA capsule | 3 mg in 3 mL | 12 mg  (3 4-mg tablets) |
| **Day 2** | placebo aprepitant capsule | 8 mg  (2 4-mg tablets) | 80 mg |  | 8 mg  (2 4-mg tablets) |
| **Day 3** | placebo aprepitant capsule | 8 mg  (2 4-mg tablets) | 80 mg |  | 8 mg  (2 4-mg tablets) |
| **Day 4** |  | 8 mg  (2 4-mg tablets) |  |  | 8 mg  (2 4-mg tablets) |

- NEPA and APR (and corresponding placebos) were administered 60 min prior to chemotherapy in the clinic/hospital on day 1, while GRAN (and corresponding placebo) and DEX were administered 30 min prior to chemotherapy on day 1.
- On days 2-3, APR was administered 24 and 48h after its administration on day 1.
- On days 2-4, DEX was administered 24, 48 and 72h after its administration on day 1.
- Due to increased exposure to dexamethasone when co-administered with netupitant and aprepitant, both CYP3A4 inhibitors, the standard dose of dexamethasone was reduced in both treatment arms.

APR, aprepitant; GRAN, granisetron; DEX, dexamethasone; NETU, netupitant, PALO, palonosetron; IV, intravenous
