# Supplementary material for: Efficacy of NEPA, a fixed antiemetic combination of netupitant and palonosetron, vs a 3‐day aprepitant regimen for prevention of chemotherapy‐induced nausea and vomiting (CINV) in Chinese patients receiving highly emetogenic chemotherapy (HEC) in a randomized Phase 3 study
Source: Cancer Med. 2020 May 30;9(14):5134–42. doi: 10.1002/cam4.3123 (PMC7367622; doi:10.1002/cam4.3123)
Supplement: Supplementary file 2 — Supplementary Material [file CAM4-9-5134-s002.docx]

**STUDY PROTOCOL SYNOPSIS**

| **Name of active ingredient** | Netupitant and palonosetron (as a fixed dose combination) (NEPA) |
| --- | --- |
| **Title of study** | A randomized, double-blind, double-dummy, parallel group, international multicenter study assessing the efficacy and safety of a NEPA compared to an extemporary combination of granisetron and aprepitant on the prevention of highly emetogenic chemotherapy-induced nausea and vomiting in patients with cancer. |
| **Study centers** | Overall 45 sites are foreseen to participate (30 sites in China, 6 in Taiwan, 3 in Thailand and 6 in South Korea).  Note: the number of sites and countries may change if required to comply with  the purpose of the study. |
| **Phase of development** | Phase 3 |
| **Objectives** | **Primary Objective：**  To demonstrate the non-inferiority of NEPA versus an extemporary combination of aprepitant and granisetron (APR/GRAN) in the prevention of highly emetogenic chemotherapy (HEC) -induced nausea and vomiting.  **Secondary Objective:**  To describe the efficacy of NEPA versus APR/GRAN by the evaluation of further secondary efficacy variables during the acute (0-24 hours), the delayed (25-120 hours) and overall (0-120 hours) phases.  To assess the safety and tolerability of NEPA in patients receiving HEC. |
| **Study design** | International multi-center, randomized, double-blind, double-dummy, parallel group stratified (by gender) study to investigate the non-inferiority of a single dose of NEPA on the prevention of nausea and vomiting compared with APR/GRAN administered prior to HEC. |
| **Number /type of subjects** | 832 randomized adult chemotherapy naïve patients (i.e. no history of cytotoxic chemotherapy) with histologically or cytologically confirmed solid tumors who are scheduled to receive cisplatin-based HEC |
| **Inclusion criteria** | For inclusion in the study, patients must fulfill all of the following criteria:   1. Provide written informed consent; 2. Male or female, aged 18 years or over; 3. Cytotoxic chemotherapy naïve; 4. Have a histologically or cytologically confirmed solid tumor malignancy; 5. Be scheduled to receive the first course of cisplatin-based chemotherapy regimen (≥ 50 mg/m^2^) that is to be administered over 1 to 4 hours on Day 1 (either alone or in combination with other chemotherapy agents); 6. Have an Eastern Cooperative Oncology Group (ECOG) performance status of 0, 1, or 2; 7. Non-fertile patient or fertile patient (male or female) using reliable contraceptive measures^1^ 8. Female patients of childbearing potential^2^ must have a negative urine pregnancy test^3^); 9. Able to read, understand, and follow the study procedures and able to complete patient diary independently. |

1Reliable contraceptive measures, for patients or sexual partners, include: implants, injectables, combined oral contraceptives, intrauterine devices, vasectomized / sterilized partner or sexual abstinence

2 Non-childbearing potential is defined as post-menopausal for at least 1 year or documented surgical sterilization or hysterectomy at least 3 months before study start.

3To be performed and verified at screening visit (Visit 1) and before study drug administration on the day chemotherapy is given (Visit 2)

| **Exclusion criteria** | Any of the following is regarded as a criterion for exclusion from the study:   1. Current use of illicit drugs or current evidence of alcohol abuse; 2. Scheduled to receive moderately emetogenic chemotherapy (MEC) or HEC from Day 2 to Day 5 following cisplatin-based chemotherapy administration; 3. Scheduled to receive bone marrow or stem cell transplant; 4. Moderately- or highly-emetogenic radiotherapy within 1 week prior to Day 1 or scheduled for study Days 1 to 5; 5. Any drug with potential antiemetic efficacy taken within 24 hours prior to Day 1; 6. Systemic corticosteroid therapy (including but not limited to dexamethasone, hydrocortisone, methylprednisolone, or prednisolone) other than that required by the protocol given within 72 hours prior to Day 1 (Note: topical or inhaled steroids are permitted); 7. Neurokinin (NK)_1_ receptor antagonists or any investigational drugs taken within 4 weeks prior to Day 1; 8. Hematologic and metabolic status inadequate for receiving a cisplatin-based HEC regimen, including any of the following criteria: 9. Absolute neutrophil count <1500/mm^3^ and white blood cell (WBC) count <3000/mm^3^ 10. Platelet count <100,000/mm^3^ 11. Bilirubin >1.5 x upper limit of normal (ULN) 12. Liver enzymes:   In patients without known liver metastases:   - 1. aspartate aminotransferase (AST) ≥ 2.5 x ULN   2. alanine aminotransferase (ALT) ≥ 2.5 x ULN   In patients with known liver metastases:   - 1. AST ≥ 5.0 x ULN   2. ALT ≥ 5.0 x ULN  1. Serum creatinine ≥ 1.5 mg/dL (standard units: ≥132.6 µMOL/L) 2. Creatinine clearance ≤ 50 mL/min; 3. Active infection (e.g . pneumonia) or any uncontrolled disease (e.g . diabetic ketoacidosis or gastrointestinal obstruction) that, in the opinion of the Investigator, may confound the results of the study or pose unwarranted risk in administering the study drug treatments; 4. History or predisposition to cardiac conduction abnormalities (like Torsade de Point, long QT syndrome or others), except for incomplete right bundle branch block; 5. Serious cardiovascular diseases, including acute myocardial infarction, unstable angina pectoris, significant valvular or pericardial disease, history of ventricular tachycardia, symptomatic chronic heart failure (New York Heart Association [NYHA] class III-IV), and severe uncontrolled arterial hypertension; 6. History of any illness that, in the opinion of the Investigator, may confound the results of the study or pose unwarranted risk in administering the study treatments 7. Any vomiting, retching, or more than mild nausea within 24 hours prior to Day 1; 8. Ongoing or recent history of somatic disease causing nausea or vomiting; 9. Symptomatic primary or metastatic central nervous system malignancy; 10. Chronic use of any CYP3A4 substrates or inhibitors (e.g . terfenadine, cisapride, astemizole, clarithromycine, ketoconazole or itraconazole) or their intake within 1 week prior to Day 1; 11. Chronic use of any CYP3A4 inducers (e.g . barbiturates, rifampicin, rifabutin, phenytoin or carbamazepine) or their intake within 4 weeks prior to Day 1; 12. Concurrent medical condition that would delay dexamethasone administration by 4 days (e.g. systemic fungal infection or uncontrolled diabetes); 13. Known contraindications to NK1 receptor antagonists, 5-HT3 receptor antagonists or dexamethasone; 14. Enrolment in a previous study with netupitant (either alone or in combination with palonosetron). |
| --- | --- |
| **Study** | **Test drug:** |
| **treatments** | Oral administration of NEPA (containing 300 mg netupitant and |
|  | 0.5 mg palonosetron) on Day 1 (with an adjusted dexamethasone regimen: |
|  | 12 mg on Day 1 + 8 mg daily from Day 2 to 4). |
|  | **Active Control:** |
|  | Oral aprepitant 125 mg (on Day 1) + 80 mg daily (on Day 2 and Day 3) and |
|  | 3 mg intravenous (IV) granisetron on Day 1 (with an adjusted dexamethasone |
|  | regimen: 12 mg on Day 1 + 8 mg daily from Day 2 to 4). |

| **Study treatments (cont.)** | Following the instruction of the interactive web response randomization system (IWRS) / interactive voice response system (IVRS) and according to the actual kit content, an unblinded study pharmacist or unblinded designated person at each study site will be responsible to prepare syringe for granisetron /saline IV infusion and provide it to the Investigator in a blinded  fashion for administration to the patients. |
| --- | --- |
| **Study duration** | Each patient will stay on study for a maximum of 28 days (including up to a  7-day screening period, 6 days on study [including 4 days on active treatment] and a follow-up visit [or a telephone call] 15 days after Day 6). |
| **Study assessments** | **Efficacy Assessments:**  Time related efficacy study parameters assessment will start at “time 0”, defined as the start time of cisplatin-based chemotherapy administration*.* Primary efficacy endpoint:  The primary efficacy endpoint is the complete response (CR, defined as no emetic episodes and no rescue medication) within 120 hours after the start of the administration of cisplatin-based HEC.  Secondary efficacy endpoints:   - CR for the 0-24 hours, 25-120 hours interval and for each 24-hour interval after the start of cisplatin-based HEC; - Absence of significant nausea (visual analogue scale [VAS] <25 mm); considering each 24-hour interval (starting from 0-24 hours from the start of cisplatin-based chemotherapy administration) as well as over 0-120 hours and 25-120 hours; - Absence of nausea (VAS <5 mm); considering each 24-hour interval (starting from 0-24 hours from the start of cisplatin-based chemotherapy administration) as well as over 0-120 hours and 25-120 hours; - Absence of emesis; considering each 24-hour interval (starting from 0-24 hours from the start of cisplatin-based chemotherapy administration) as well as over 0-120 hours and 25-120 hours; - Absence of rescue medication use; considering each 24-hour interval (starting from 0-24 hours from the start of cisplatin-based chemotherapy administration) as well as over 0-120 hours and 25-120 hours; - Severity of nausea (measured by means of a VAS) for each 24-hour interval; |

| **Study assessments (cont.)** | - Time to first emetic episode, time to first rescue medication intake, time to treatment failure (based on time to the first emetic episode or time to the first rescue medication intake, whichever occurs first); - Impact on patients’ daily life activities in the acute and delayed phase following the administration of cisplatin-based chemotherapy as assessed by the Functional Living Index - Emesis (FLIE) questionnaire.   **Safety:**  Safety assessments will comprise the recording and assessing all adverse events (AEs) /serious adverse events (SAEs); laboratory evaluations (hematology, blood chemistry and urinalysis); periodic measurement of vital signs and electrocardiogram (ECG); and the performance of physical  examinations. |
| --- | --- |
